# NGS-based phylogeny of diphtheria-related pathogenicity factors in different *Corynebacterium spp.* implies species-specific virulence transmission

**DOI:** 10.1186/s12866-019-1402-1

**Published:** 2019-02-01

**Authors:** Alexandra Dangel, Anja Berger, Regina Konrad, Andreas Sing

**Affiliations:** 1 Bavarian Health and Food Safety Authority, 85764 Oberschleissheim, Germany; 2 German National Consiliary Laboratory on Diphtheria, 85764 Oberschleissheim, Germany

**Keywords:** Bacterial infections, Diphtheria, Whole genome sequencing, Public health, Corynebacterium, Pathogenicity, Phylogeny, Diphtheria toxin, Prophages, Genomic Islands

## Abstract

**Background:**

Diphtheria toxin (DT) is produced by toxigenic strains of the human pathogen *Corynebacterium diphtheriae* as well as zoonotic *C. ulcerans* and *C. pseudotuberculosis*. Toxigenic strains may cause severe respiratory diphtheria, myocarditis, neurological damage or cutaneous diphtheria. The DT encoding *tox* gene is located in a mobile genomic region and *tox* variability between *C. diphtheriae* and *C. ulcerans* has been postulated based on sequences of a few isolates. In contrast, species-specific sequence analysis of the diphtheria toxin repressor gene (*dtxR*), occurring both in toxigenic and non-toxigenic *Corynebacterium* species, has not been done yet. We used whole genome sequencing data from 91 toxigenic and 46 non-toxigenic isolates of different pathogenic *Corynebacterium* species of animal or human origin to elucidate differences in extracted DT, DtxR and *tox*-surrounding genetic elements by a phylogenetic analysis in a large sample set.

**Results:**

Sequences of both DT and DtxR, extracted from whole genome sequencing data, could be classified in four distinct, nearly species-specific clades, corresponding to *C. diphtheriae*, *C. pseudotuberculosis*, *C. ulcerans* and atypical *C. ulcerans* from a non-toxigenic toxin gene-bearing wildlife cluster. Average amino acid similarities were above 99% for DT and DtxR within the four groups, but lower between them. For DT, subgroups below species level could be identified, correlating with different *tox*-comprising mobile genetic elements. In most *C. diphtheriae*, *tox* genes were located within known prophages. In contrast, in *C. ulcerans* diverse *tox*-including mobile elements could be identified: either prophages differing from *C. diphtheriae* prophages or an alternative pathogenicity island (PAI) described previously. One isolate showed a different, shorter *tox*-comprising putative PAI. Beyond the *tox*-overlapping elements, most isolates harbored a variety of additional prophages.

**Conclusion:**

Our NGS data from 137 isolates indicate the existence of different genetic backgrounds of DT-mediated pathogenicity in different *Corynebacterium* species and evolution of once acquired pathogenicity features with the strains. Different groups of pathogenicity-related elements within *C. ulcerans* imply that *tox* transmission pathways between isolates may differ in the zoonotic species and contribute to their emerging pathogenic potential.

**Electronic supplementary material:**

The online version of this article (10.1186/s12866-019-1402-1) contains supplementary material, which is available to authorized users.

## Background

Besides several non-pathogenic species, the genus *Corynebacterium* comprises more than 50 medically relevant ones. Some of them have a high pathogenic potential for human and animal hosts [1, 2]. An important pathogen is *Corynebacterium diphtheriae*, with humans as their almost exclusive reservoir and only very rare case reports of isolates from infected animals, such as e.g. horses or pigs [3, 4]. Additional important pathogenic species include zoonotic *C. ulcerans* and *C. pseudotuberculosis*. Despite the omnipresence of vaccination programs against toxoids in most developed countries, diseases with toxigenic corynebacteria occur consistently, due to various reasons: For example vaccination gaps and/or global travel activity from and to endemic countries occur [5]. Also other factors, for example quality and availability of vaccines may be problematic, especially in low-income countries [6]. In addition to classic *C. diphtheriae* mediated diseases, infections with toxigenic *C. ulcerans* outnumber those of toxigenic *C. diphtheriae* since several years in central Europe. *C. ulcerans* is increasingly recognized as emerging pathogen with inland animal contact as the most important risk factor [7, 8] in contrast to most often abroad acquired infections by toxigenic *C. diphtheriae*. *C. diphtheriae*, as well as *C. ulcerans* and *C. pseudotuberculosis* are all able to produce various virulence- and pathogenicity-related proteins, which are often associated with cell adhesion or iron homeostasis [9]. The diphtheria toxin (DT) encoding *tox* gene-bearing strains may produce DT, which is one of the most potent bacterial toxins. Thus, toxigenic strains may cause severe respiratory diphtheria, myocarditis, neurological damage or severe cutaneous diphtheria. DT was one of the first bacterial toxins detected [10] and its mode of action and structure have been extensively studied [11]. It is an AB exotoxin, acting by inhibition of protein synthesis. It consists of a catalytic subunit A and an adhesion- and cell entry-mediating subunit B with a receptor-binding (R) and a translocation-mediating (T) domain [12]. The DT encoding *tox* gene is located in a mobile genomic region. In *C. diphtheriae* different *tox* gene-carrying prophages, introduced into the bacterial genome through lysogenic conversion, have already been described and sequenced [9, 13]. In *C. ulcerans* an alternative pathogenicity island (PAI) has been detected additionally [5]. Genetic *tox* variability within the species *C. diphtheriae* [14] or between single cases of *C. diphtheriae* and *C. ulcerans* has been shown [15]. However, it is not known yet if surrounding prophages or PAIs are absolutely species-specific or also transmitted between *Corynebacterium* species. DT is furthermore part of an iron-dependent regulation system with the diphtheria toxin repressor (DtxR) acting as main regulatory control center on DT, but also on various other virulence factors [16]. In contrast to the DT encoding *tox* gene, the *dtxR* gene occurs both in toxigenic and non-toxigenic isolates of the different *Corynebacterium* species and its genetic variability between them has not been analysed in detail yet.

During the last years the German Consiliary Laboratory on Diphtheria has sequenced a substantial number of genomes of *Corynebacterium spp.* isolates by Next Generation Sequencing (NGS), mainly in regard to various outbreak investigations. In this study we used NGS-generated *Corynebacterium spp*. isolate genomes, partly from previous outbreak investigations, to analyse the differences between several pathogenic *Corynebacterium* species regarding DT, its regulator DtxR and the *tox* gene-surrounding mobile elements. We aimed to investigate if differences between species can be observed on a regular basis when a larger sample set is analysed and which phylogenetic conclusions regarding the DT-mediated pathogenicity of medically relevant corynebacteria can be drawn from the results.

## Results

To investigate the potential differences of DT, its iron-dependent regulator DtxR and the *tox* gene-surrounding, pathogenicity-related genetic elements in pathogenic corynebacteria, we conducted WGS analyses with isolates from different *Corynebacterium* and host species and from different geographic backgrounds (Table 1).

**Table 1:** Isolates and epidemiology

Sample name	Species	Year	Host	Host Disease	Risk factor	Toxin-PCR	Elek	Outbreak ref
08–1143-CB1	*C. ulcerans*	2007	Pig	Asymptomatic colonisation	NA	Positive	Positive	[7]
KL0126-cb2	*C. ulcerans*	2007	Human	Diphtheria-like	Animal contact (pig)	Positive	Positive	[7]
KL0160	*C. pseudotuberculosis*	2009	Goat	NA	NA	Negative	N.a.	–
KL0182	Atypical *C. ulcerans* (NTTB)	2010	Boar	Lymphadenitis	NA	Positive	Negative	–
KL0183	Atypical *C. ulcerans* (NTTB)	2010	Boar	Lymphadenitis	NA	Positive	Negative	–
KL0194	*C. ulcerans*	2010	Human	NA	NA	Positive	Positive	–
KL0195	*C. ulcerans* (NTTB)	2010	Human	NA	NA	Positive	Negative	–
KL0199	*C. ulcerans*	2010	Human	NA	NA	Negative	N.a.	–
KL0246-cb3	*C. ulcerans*	2010	Human	NA	Animal contact (cat)	Positive	Positive	[7]
KL0251-cb4	*C. ulcerans* (NTTB)	2010	Cat	Asymptomatic colonisation	NA	Positive	Negative	[7]
KL0252-cb5	*C. ulcerans* (NTTB)	2010	Cat	Asymptomatic colonisation	NA	Positive	Negative	[7]
KL0259	Atypical *C. ulcerans* (NTTB)	2011	Boar	Lung abscess	NA	Positive	Negative	–
KL0260	Atypical *C. ulcerans* (NTTB)	2011	Boar	Multiple abscesses	NA	Positive	Negative	–
KL0262	*C. pseudotuberculosis* (NTTB)	2011	Buffalo	NA	NA	Positive	Negative	–
KL0263	*C. pseudotuberculosis* (NTTB)	2011	Buffalo	NA	NA	Positive	Negative	–
KL0264	*C. pseudotuberculosis*	2011	Human	NA	NA	Negative	Negative	–
KL0265	*C. pseudotuberculosis*	2011	Human	NA	NA	Negative	Negative	–
KL0266	*C. pseudotuberculosis*	2011	Human	NA	NA	Negative	Negative	–
KL0269	*C. pseudotuberculosis*	2011	Goat	Abscess	NA	Negative	N.a.	–
KL0276	*C. diphtheriae*	2011	Human	NA	NA	Positive	Positive	–
KL0315-cb6	*C. ulcerans* (NTTB)	2012	Human	Ulcer	Animal contact (dog)	Positive	Negative	[7]
KL0318-cb7	*C. ulcerans* (NTTB)	2012	Dog	Asymptomatic colonisation	NA	Positive	Negative	[7]
KL0330	*C. diphtheriae*	2012	Human	Wound infection	Homeless	Negative	N.a.	[17]
KL0345	*C. ulcerans* (NTTB)	2012	Cat	Rhinitis	NA	Positive	Negative	–
KL0349	*C. ulcerans*	2012	Human	NA	NA	Negative	Negative	–
KL0350	*C. ulcerans*	2012	Human	NA	NA	Positive	Positive	–
KL0355	*C. diphtheriae*	2012	Human	Wound infection	NA	Negative	N.a.	[17]
KL0360	*C. diphtheriae*	2012	Human	Wound infection	Travel (Africa)	Negative	N.a.	[17]
KL0371	*C. diphtheriae*	2012	Human	Wound infection	NA	Negative	N.a.	[17]
KL0372	*C. diphtheriae*	2012	Human	Wound infection	Mixed infection (*S. aureus*, *Enterobacter spp., Pantoea spp*.)	Negative	N.a.	[17]
KL0374	Atypical *C. ulcerans* (NTTB)	2012	Boar	NA	NA	Positive	Negative	–
KL0377	*C. diphtheriae*	2012	Human	Wound infection	Mixed infection (hemolysing *Streptococcus spp*., group A)	Negative	N.a.	[17]
KL0382	Atypical *C. ulcerans* (NTTB)	2012	Boar	NA	NA	Positive	Negative	–
KL0386	Atypical *C. ulcerans* (NTTB)	2012	Boar	NA	NA	Positive	Negative	–
KL0387-cb8	*C. ulcerans* (NTTB)	2012	Human	Wound infection	Animal contact (cat)	Positive	Negative	[7]
KL0392-cb9	*C. ulcerans* (NTTB)	2012	Cat	Asymptomatic colonisation	NA	Positive	Negative	[7]
KL0394	Atypical *C. ulcerans* (NTTB)	2013	Boar	NA	NA	Positive	Negative	–
KL0395	Atypical *C. ulcerans* (NTTB)	2013	Boar	NA	NA	Positive	Negative	–
KL0396	Atypical *C. ulcerans* (NTTB)	2013	Boar	NA	NA	Positive	Negative	–
KL0400	Atypical *C. ulcerans* (NTTB)	2013	Boar	Abscess	NA	Positive	Negative	–
KL0401	Atypical *C. ulcerans* (NTTB)	2013	Boar	Abscess	NA	Positive	Negative	–
KL0433	*C. ulcerans*	2013	Human	Wound infection	NA	Positive	Positive	–
KL0434	*C. diphtheriae*	2013	Human	Wound infection (insect bite)	Travel (destination not reported)	Negative	N.a.	[17]
KL0438	*C. diphtheriae*	2013	Human	Wound infection	NA	Negative	N.a.	[17]
KL0442	*C. ulcerans* (NTTB)	2013	Human	Ulcer	NA	Positive	Negative	–
KL0451	*C. ulcerans*	2013	Human	Sepsis, infected dialysis shunt	NA	Negative	N.a.	–
KL0459	*C. ulcerans*	2013	Human	Diphtheria-like	Animal contact (dog)	Positive	Positive	–
KL0461	*C. diphtheriae*	2013	Human	Wound infection	NA	Negative	N.a.	[17]
KL0468	*C. ulcerans* (NTTB)	2013	Human	NA	NA	Positive	Negative	–
KL0472	*C. ulcerans (*NTTB)	2013	Human	NA	NA	Positive	Negative	–
KL0475	*C. ulcerans*	2013	Human	Wound infection	NA	Positive	Positive	–
KL0476	*C. diphtheriae*	2013	Human	Wound infection	Travel (destination not reported)	Negative	N.a.	[17]
KL0479	*C. diphtheriae*	2013	Human	Wound infection	Hepatitis C	Negative	N.a.	[17]
KL0483	*C. ulcerans*	2014	Human	Wound infection	Animal contact (dog)	Positive	Positive	–
KL0497	*C. ulcerans*	2014	Human	Wound infection	NA	Positive	Positive	–
KL0501	*C. ulcerans* (NTTB)	2014	Human	Pus	NA	Positive	Negative	–
KL0507	*C. diphtheriae*	2014	Human	Sepsis	NA	Negative	N.a.	[17]
KL0515	*C. ulcerans* (NTTB)	2014	Human	Wound infection	NA	Positive	Negative	–
KL0522	*C. diphtheriae*	2014	Human	Wound infection	Mixed infection (*S. pyogenes*), drug abuse	Negative	N.a.	[17]
KL0540	*C. ulcerans*	2014	Human	Wound infection	NA	Positive	Positive	–
KL0541	*C. ulcerans* (NTTB)	2014	Human	Diphtheria-like	NA	Positive	Negative	–
KL0547	*C. ulcerans*	2014	Human	Wound infection	Chronic wound with *C. ulcerans*, *Streptococcus spp*. (C), *S. aureus*	Positive	Positive	–
KL0556	*C. ulcerans*	2014	Human	Wound infection	NA	Positive	Positive	–
KL0557	*C. diphtheriae*	2014	Human	Wound infection	Travel (Sri Lanka)Mixed infection (*S. pyogenes*)	Negative	N.a.	[17]
KL0565	*C. diphtheriae*	2014	Human	Phlegmon	NA	Positive	Positive	–
KL0581	Atypical *C. ulcerans* (NTTB)	2014	Boar	Abscess	NA	Positive	Negative	–
KL0585	*C. diphtheriae*	2015	Human	Wound infection, deep wound	NA	Negative	N.a.	[17]
KL0598	Atypical *C. ulcerans* (NTTB)	2015	Boar	Lymph node abscess	NA	Positive	Negative	–
KL0599	*C. diphtheriae*	2015	Human	Wound infection, abscess	Homeless	Negative	N.a.	[17]
KL0603	*C. diphtheriae*	2015	Human	Abscess	Travel (Sri Lanka)	Positive	Positive	[5]
KL0613	*C. diphtheriae*	2015	Human	Ulcer	NA	Positive	Positive	[5]
KL0615	Atypical *C. ulcerans* (NTTB)	2015	Boar	Abscess	NA	Positive	Negative	–
KL0623	*C. diphtheriae*	2015	Human	Wound infection	Travel (asylum seeker Eritrea)	Positive	Positive	[5]
KL0625	*C. diphtheriae*	2015	Human	Ulcer	Travel (asylum seeker Ethiopia), pulmonary tuberculosis	Positive	Positive	[5]
KL0631	*C. diphtheriae*	2015	Human	Abscess	Travel (asylum seeker)	Positive	Positive	[5]
KL0633	*C. diphtheriae*	2015	Human	Wound infection	Travel (asylum seeker Somalia)	Positive	Positive	[5]
KL0638	*C. diphtheriae*	2015	Human	Tonsilitis, pharyngitis, diptheria-like	Travel (Thailand)animal contact (cat, rabbit)	Positive	Positive	[5]
KL0652	*C. diphtheriae*	2015	Human	Abscess	Travel (asylum seeker)	Positive	Positive	[5]
KL0654	*C. diphtheriae*	2015	Human	NA	Travel (asylum seeker Eritrea)	Positive	Positive	[5]
KL0655	*C. diphtheriae*	2015	Human	NA	Travel (asylum seeker)	Positive	Positive	[5]
KL0663	*C. diphtheriae*	2015	Human	Wound infection	Travel (asylum seeker Eritrea)	Positive	Positive	[5]
KL0670	*C. diphtheriae*	2015	Human	NA	Travel (asylum seeker)	Positive	Positive	[5]
KL0675	*C. diphtheriae*	2015	Human	Wound infection, phlegmon, sepsis	Homeless	Negative	N.a.	[17]
KL0676	*C. diphtheriae*	2015	Human	Wound infection	NA	Negative	N.a.	[17]
KL0678	*C. diphtheriae*	2015	Human	Wound infection	Homeless, alcohol abuse	Negative	N.a.	[17]
KL0691	*C. diphtheriae*	2015	Human	Wound infection	Homeless	Negative	N.a.	[17]
KL0693	*C. diphtheriae*	2015	Human	Sepsis, pneumonia	Origin: Poland	Negative	N.a.	[17]
KL0698	*C. diphtheriae*	2015	Human	Wound infection	NA	Negative	N.a.	[17]
KL0707	Atypical *C. ulcerans* (NTTB)	2015	Boar	NA	NA	Positive	Negative	–
KL0709	Atypical *C. ulcerans* (NTTB)	2015	Boar	NA	NA	Positive	Negative	–
KL0713	*C. diphtheriae*	2015	Human	Wound infection, deep wound	NA	Negative	N.a.	[17]
KL0747	*C. diphtheriae*	2016	Human	Wound infection	Homeless, drug abuse	Negative	N.a.	[17]
KL0759	*C. diphtheriae*	2016	Human	Olecranon bursitis	Homeless	Negative	N.a.	[17]
KL0762	*C. diphtheriae*	2016	Human	Pharyngitis, tonsillitis	NA	Negative	N.a.	[17]
KL0768	*C. diphtheriae*	2016	Human	Sepsis, endocarditis	Homeless	Negative	N.a.	[17]
KL0770	*C. diphtheriae*	2016	Human	Wound infection, ulcer	NA	Negative	N.a.	[17]
KL0773	Atypical *C. ulcerans* (NTTB)	2016	Boar	Abscess	NA	Positive	Negative	–
KL0774	Atypical *C. ulcerans* (NTTB)	2016	Boar	Abscess	NA	Positive	Negative	–
KL0785	*C. ulcerans*	2016	Human	Wound infection, diabetic foot	Animal contact (dog),	Positive	Positive	–
KL0788	*C. diphtheriae*	2016	Human	phlegmon (post human bite)	Origin: Poland, homeless	Negative	N.a.	[17]
KL0796	*C. ulcerans*	2016	Human	Wound infection	NA	Positive	Positive	–
KL0798	*C. diphtheriae*	2016	Human	Wound infection, burn	Homeless	Negative	N.a.	[17]
KL0811	*C. diphtheriae*	2016	Human	Wound infection	Origin: Poland, homeless, alcohol abuse	Negative	N.a.	[17]
KL0812	*C. diphtheriae*	2016	Human	Wound infection, abscess	Homeless	Negative	N.a.	[17]
KL0818	*C. ulcerans* (NTTB)	2016	Human	ulcer	Diabetic foot	Positive	Negative	–
KL0825	*C. ulcerans*	2016	Human	Wound infection	NA	Positive	Positive	–
KL0832	*C. ulcerans*	2016	Human	Wound infection	NA	Positive	Positive	–
KL0840	*C. ulcerans*	2016	Human	Eczema, Erysipelas	Animal contact (cat)	Positive	Positive	–
KL0846	*C. ulcerans* (NTTB)	2016	Human	Ulcer	Diabetic foot	Positive	Negative	–
KL0853	*C. ulcerans*	2016	Human	Wound infection (post dog bite)	Animal contact (dog)	Negative	N.a.	–
KL0867	*C. ulcerans* (NTTB)	2016	Human	Wound infection	NA	Positive	Negative	–
KL0870	*C. ulcerans* (NTTB)	2016	Human	Eczema	NA	Positive	Negative	–
KL0876	*C. ulcerans*	2017	Human	Wound infection	NA	Negative	N.a.	–
KL0880	*C. ulcerans* (NTTB)	2017	Human	Ulcer	NA	Positive	Negative	–
KL0882	Atypical *C. ulcerans* (NTTB)	2017	Boar	NA	NA	Positive	Negative	–
KL0883	Atypical *C. ulcerans* (NTTB)	2017	Boar	Abscess	NA	Positive	Negative	–
KL0884	Atypical *C. ulcerans* (NTTB)	2017	Boar	Abscess	NA	Positive	Negative	–
KL0886	Atypical *C. ulcerans* (NTTB)	2017	Boar	NA	NA	Positive	Negative	–
KL0887	Atypical *C. ulcerans* (NTTB)	2017	Boar	Abscess	NA	Positive	Negative	–
KL0927	*C. diphtheriae*	2017	Human	Wound infection	Homeless	Negative	N.a.	–
KL0938	Atypical *C. ulcerans* (NTTB)	2017	Boar	Abscess	NA	Positive	Negative	–
KL0941	*C. ulcerans*	2017	Human	Wound infection	NA	Negative	N.a.	–
KL0950	*C. diphtheriae*	2017	Human	Wound infection	Travel (Thailand)Mixed infection (*S. pyogenes*)	Positive	Positive	–
KL0956	*C. diphtheriae*	2017	Human	Wound infection	NA	Positive	Positive	–
KL0957	Atypical *C. ulcerans* (NTTB)	2017	Boar	Abscess	NA	Positive	Negative	–
KL0968	Atypical *C. ulcerans* (NTTB)	2017	Boar	Abscess	NA	Positive	Negative	–
KL1003	Atypical *C. ulcerans* (NTTB)	2017	Boar	Abscess	NA	Positive	Negative	–
KL1006	Atypical *C. ulcerans* (NTTB)	2017	Boar	Abscess	NA	Positive	Negative	–
KL1007	Atypical *C. ulcerans* (NTTB)	2017	Boar	Abscess	NA	Positive	Negative	–
KL1008	Atypical *C. ulcerans* (NTTB)	2017	Boar	Abscess	NA	Positive	Negative	–
KL1009	Atypical *C. ulcerans* (NTTB)	2017	Boar	Abscess	NA	Positive	Negative	–
KL1010	Atypical *C. ulcerans* (NTTB)	2017	Boar	Abscess	NA	Positive	Negative	–
KL1015	*C. ulcerans* (NTTB)	2017	Human	Carrier status	Animal contact (cat, dog)	Positive	Negative	–
KL1017	*C. ulcerans*	2017	Cattle	Milk isolate, mastitis	NA	Negative	N.a.	–
KL1025	*C. ulcerans* (NTTB)	2017	Cat	Carrier status	NA	Positive	Negative	–
KL1058	*C. diphtheriae*	2017	Human	Wound infection	Travel (Angola), war victim	Negative	N.a.	–
KL1059	*C. diphtheriae*	2017	Human	Wound infection	Travel (Angola), war victim	Positive	Positive	–

*NA* No information available, *N.a.* Not analysed

Analysed isolates from *C. diphtheria*e were all isolated from human hosts from different geographic regions of Germany or Switzerland, partly with travel or migration history. Those of *C. ulcerans* were obtained from human and different animal hosts, such as cats, dogs, pigs and cattle. The isolates from a group of biochemical and genetic atypical *C. ulcerans* originated from a NTTB wildlife cluster of wild boar and roe deer (*unpublished observations*) and those of *C. pseudotuberculosis* from humans, buffalo or goats (Table 1). Several of the *C. diphtheriae* and *C. ulcerans* isolates belonged to different previously identified community-based or zoonotic outbreak clusters [5, 7, 17]. The NGS datasets obtained in previous outbreak analyses were bioinformatically reanalysed together with the data of currently sequenced isolates, after species identification with MALDI-TOF MS and analysis of toxin status by qPCR and by Elek test. Whole genome sequences of all 137 toxigenic and non-toxigenic isolates underwent k-mer based species confirmation. Subsequently the sequences were assembled into contigs, contigs were ordered according to corresponding reference sequences and annotated for coding sequences and other genetic elements such as rRNAs, tRNAs, repeats and additionally for known prophages.

For phylogenetic analyses of DT and its regulator DtxR, translated protein sequences of both genes were extracted from the annotated assemblies. They were aligned and finally ML phylogenies were generated and visualized in a phylogenetic tree.

In case of DT, 22 DT sequences from *C. diphtheriae* and 17 from *C. ulcerans* available on the NCBI public database were additionally included to base the initial DT comparison on more international data (Additional file 1: Table S1). These additional data originated from human hosts (*C. diphtheriae* and *C. ulcerans*) and from dogs, moles, owls (*C. ulcerans*) or unknown host organisms. For one of the sequenced toxigenic *C. diphtheriae* isolates (KL0655) the genomic region of the *tox* gene was poorly covered by sequencing reads, thus it was excluded from DT alignment and DT phylogeny analysis. Thereby, DT sequences of 90 of the 91 sequenced toxigenic and NTTB isolates were analysed together with 39 NCBI-derived DT sequences. With regard to DtxR, sequences of all 137 toxigenic or non-toxigenic isolates were phylogenetically analysed together.

**Fig. 1:**
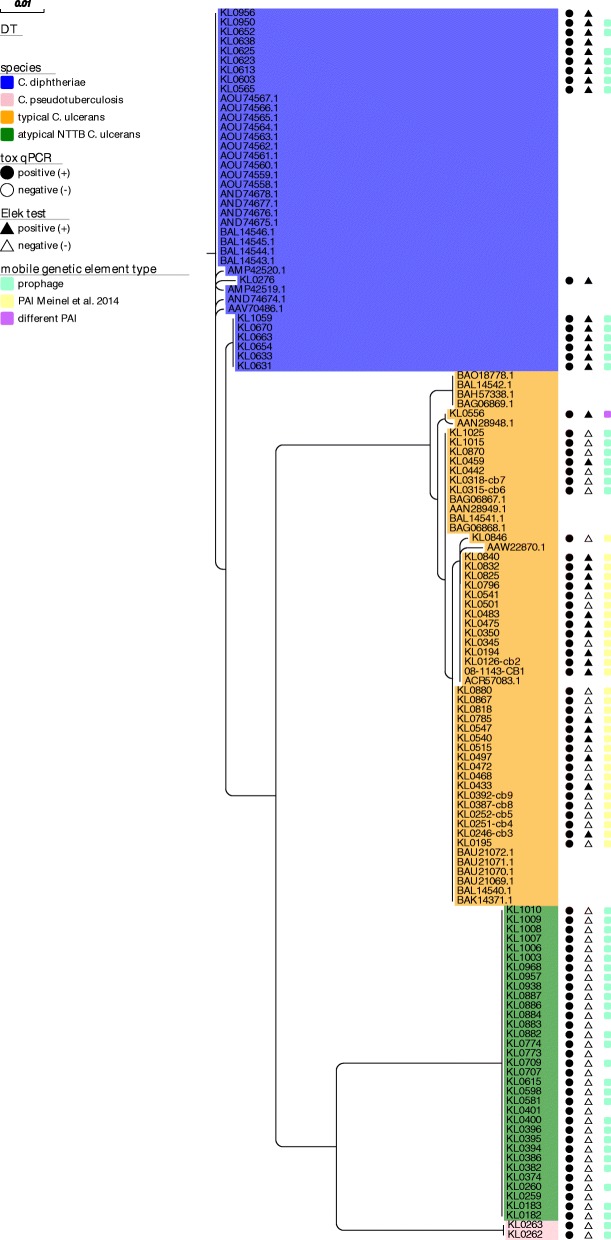
Maximum likelihood phylogeny of translated DT sequences from different *Corynebacterium* species. Translated DT sequences of 90 of 91 toxigenic *Corynebacterium spp.* isolates were extracted, aligned together with 39 NCBI derived DT sequenced from *C. diphtheriae* and *C. ulcerans* and a maximum likelihood tree was built. Species affiliation is indicated by background coloring: *C. diphtheriae* = blue, *C. pseudotuberculosis* = pink, typical *C. ulcerans* = orange, atypical NTTB *C. ulcerans* = green. Results of *tox* qPCR and Elek tests are indicated by circle and triangle shaped bars, respectively: positive = black, negative = white. The type of detected *tox*-surrounding genetic mobile element is indicated by colored boxes on the right side of the tree: prophage = turquoise, alternative PAI described in [7] = yellow, different PAI than the one described in [7] = purple

**Fig. 2:**
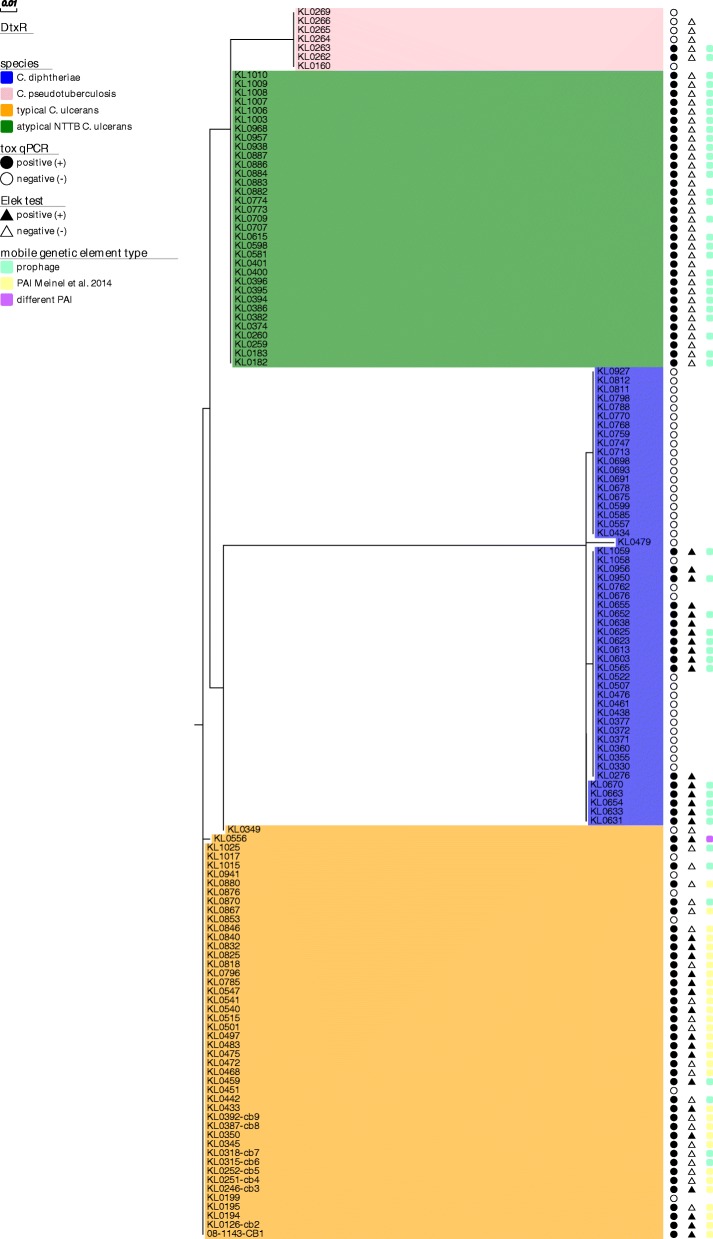
Maximum likelihood phylogeny of translated DtxR sequences from different *Corynebacterium* species. Translated DtxR sequences of all 137 toxigenic and non-toxigenic *Corynebacterium spp.* isolates were extracted, aligned and a maximum likelihood tree was built. Species affiliation is indicated by background coloring: *C. diphtheriae* = blue, *C. pseudotuberculosis* = pink, typical *C. ulcerans* = orange, atypical NTTB *C. ulcerans* = green. Results of *tox* qPCR and Elek tests are indicated by circle and triangle shaped bars, respectively: positive = black, negative = white. The type of detected *tox*-surrounding mobile genetic regions is indicated by colored boxes on the right side of the tree: prophage = turquoise, alternative PAI described in [7] = yellow, different PAI than the one described in [7] = purple

Figures 1 and 2 show that both DT and DtxR cluster in nearly species-specific distinct clades, separating the four groups of *C. diphtheria*e, *C. ulcerans*, atypical NTTB *C. ulcerans* and *C. pseudotuberculosis*.

For DT (Fig. 1) the clades of *C. pseudotuberculosis* and atypical *C. ulcerans* showed identical sequences within the clades. However, it has to be noted that the toxigenic *C. pseudotuberculosis* clade consisted of two non-representative, related isolates only, both originating from buffalo hosts from a common submitter in 2011. In contrast, minimum sequence similarity within the clades of *C. diphtheriae* and *C. ulcerans* was at 89.2 and 98.6%, respectively (Table 2A). Interestingly, the higher DT sequence variation in *C. diphtheriae* was caused by only two isolates, one human isolate from a urethral swab from our sample set (KL0276) and one downloaded from NCBI (AVV70486). In both samples the higher variation was caused by significant length and sequence variations in the DT signal peptide, which is located upstream of the A-fragment of the processed DT. However, it is not clear if these variations represent factual differences or artefacts from short read sequencing or assembly. After exclusion of the two samples, minimum DT sequence similarity was at 99.5% in *C. diphtheriae* (Table 2A).

**Table 2:**
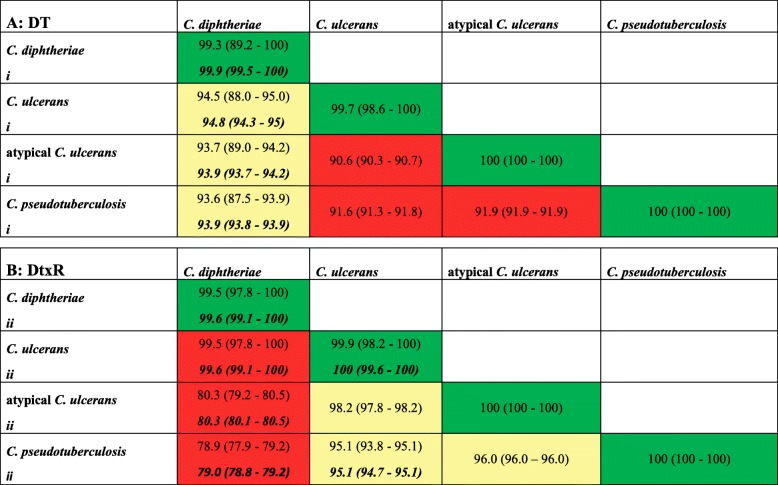
Similarity of DT (A) and DtxR (B) sequences within and between clades

Similarity percentages within and between identified DT (A) and DtxR (B) clades are shown and color-coded for high (green: > 99%), intermediate (yellow: 93 - ≤ 99%) and low (red: < 93%) similarity. Values after exclusion of samples are indicated with bold italics: i) After exclusion of *C. diphtheriae* isolates KL0276 and AVV70486. ii) After exclusion of *C. diphtheriae* belfanti isolate KL0479 and *C. ulcerans* isolate KL0349

Besides sporadic SNPs and the two samples with strongly differing signal peptide sequences, the SNPs found to differentiate between *C. diphtheriae* and *C. ulcerans* were mainly located in the receptor-binding (R) domain of the B fragment of DT, as already postulated on the basis of two Sanger sequenced *C. ulcerans* isolates and one *C. diphtheriae* reference sequence by [15]. Processed DT sequences from atypical NTTB *C. ulcerans*, however, were more similar to *C. diphtheriae*. Interestingly, the 33 atypical NTTB isolates showed also significant differences in the signal peptide sequence compared to the other clades, but identical sequences among them, whereas *C. diphtheriae*, *C. pseudotuberculosis* and typical *C. ulcerans* signal peptide sequences were very similar except for a few SNPs. Minimum DT similarity between the four clades ranged from 90.3 to 94.3% after exclusion of KL0276 and AAV70486 and mean between-clade DT similarity from 90.6 to 94.8% (Table 2A). In addition to the four main clades, several smaller branches could be identified within the two DT clades of *C. diphtheriae* and typical *C. ulcerans* based on the observed slight within-clade variations (Fig. 1).

For DtxR (Fig. 2) homogeneity within the four clades was similarly high, also in *C. diphtheriae* and regular *C. ulcerans* clades with minimum sequence similarity of 97.8 and 98.2%, respectively, although toxigenic and non-toxigenic isolates were included in the comparison of DtxR (Table 2B). The main impact on the slight within-clade variability of DtxR came from one outlier sample (KL0479) in *C. diphtheriae*, which was the only sample in the sample set of biotype belfanti. The same was true for within-clade variability in *C. ulcerans* and KL0349, a human isolate from an interlaboratory comparison. By exclusion of both samples DtxR similarities within the clades of *C. diphtheriae* and *C. ulcerans* were enhanced to ≥99.1% and ≥ 99.6%, respectively. The variability between *C. diphtheriae* and the other species was higher for DtxR than DT, whereas the variability between the other species (except *C. diphtheriae*) was higher for DT (Table 2A and B).

DT is the most important pathogenic factor within *C.diphtheriae* and is known to be embedded in a prophage in this species. After the discovery of nearly species-specific clades of DT and DtxR in the genus *Corynebacterium* we aimed to investigate if there are also species-specific differences regarding *tox*-surrounding genomic regions. We searched and annotated known prophage regions with the PHASTER software, which relies on a large database of viral and bacterial genomes to detect prophage regions in assembly data. Prophage sequences surrounding the *tox* gene could be identified for 13 of 16 WGS datasets from toxigenic *C. diphtheriae*, for both toxigenic *C. pseudotuberculosis*, for 27 of the 33 atypical NTTB *C. ulcerans*, but only in seven out of 39 toxigenic typical *C. ulcerans* isolates, respectively. Besides *tox* gene-surrounding prophage regions one to six different intact or incomplete prophage sequences were detected in both toxigenic and non-toxigenic isolates of the different *Corynebacterium* species distributed within the genomes (Additional file 2: Table S3).

Previously, we identified an additional PAI in a *C. ulcerans* strain reanalysed in this study (KL0251). The PAI started with a tRNA-Arg CDS with ACG codon, located approximately 6 kb upstream of the *tox* gene. The tRNA-Arg CDS was followed by a CDS coding for a putative integrase, another CDS carrying a Helix-turn-Helix (HTH) motif, one with a DUF955-domain suspected to be catalytically active, three coding for hypothetical proteins and two of putative integrase/transposases. The PAI was then continued with the *tox* gene and was terminated with a 100 bp sequence, repeating a part of the leading tRNA-Arg and downstream bases as a pseudo-tRNA repeat [7].

**Fig. 3:**
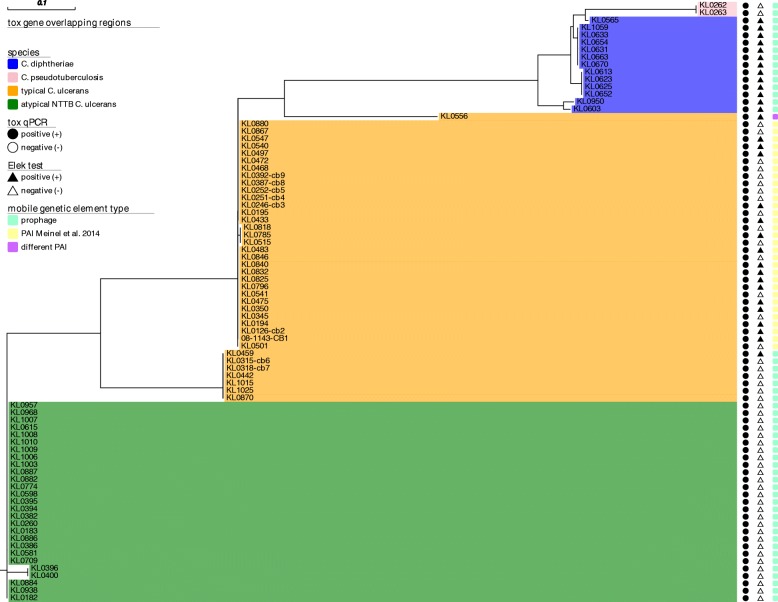
Maximum likelihood phylogeny of *tox* gene-overlapping prophage and PAI sequences from different *Corynebacterium* species. *Tox* gene-overlapping prophage and detected alternative PAI regions of 81 toxigenic *Corynebacterium spp.* isolates were aligned and a maximum likelihood tree was built. Species affiliation is indicated by background coloring: *C. diphtheriae* = blue, *C. pseudotuberculosis* = pink, typical *C. ulcerans* = orange, atypical NTTB *C. ulcerans* = green. Results of *tox* qPCR and Elek test are indicated by circle and triangle shaped bars, respectively: positive = black, negative = white. The type of detected *tox*-surrounding mobile genetic regions is indicated by colored boxes on the right side of the tree: prophage = turquoise, alternative PAI described in [7] = yellow, different PAI than the one described in [7] = purple

We searched for the existence of this PAI in all 137 NGS datasets. In 31 of the 32 toxigenic *C. ulcerans* where no prophage was detected to overlap with the *tox* gene, the PAI was present, but in none of the other *C. ulcerans* isolates or in any isolate of the other species. In each of these 31 *C. ulcerans* isolates, the sequence from the tRNA-Arg CDS to 200 bp downstream of the *tox* gene was extracted, aligned and used for a ML phylogeny together with the *tox*-overlapping prophage sequences detected in the other isolates (Fig. 3). The ML tree of the pathogenicity-related *tox* gene-surrounding nucleotide regions again classified the isolates in the same nearly species-specific groups as for DT and DtxR. However, in *C. diphtheriae* prophage sequences showed some extent of heterogeneity (Fig. 3, blue clade). They divided in several smaller branches. The first branch included KL0603 and KL0950. The second branch consisted of KL0613, KL0623, KL0625 and KL0652. The third branch included KL0654, KL0633, KL0631, KL0663, KL070 and KL1059 and the fourth only the single sample KL0565, located at a common node with the prophages of *C. pseudotuberculosis*.

*Tox*-overlapping phages of the two related *C. pseudotuberculosis* isolates were identical as expected (Fig. 3, pink clade). However, only in one of them an additional phage was detected, which was not located surrounding the *tox* gene (Additional file 2: Table S3).

In typical *C. ulcerans* it became obvious by substantially distinct branches that the sequences from the *tox* gene-surrounding prophages clearly differed from the extracted alternative PAIs (Fig. 3, orange clade) and both were independent from the underlying host species. In 28 of the 31 *C. ulcerans* samples the identified PAI had a length of 7.7 kb and the extracted sequences were very homogeneous. Three additional isolates (KL0515, KL0785, KL0818) of common MLST type 514 were obtained from wound infections from human patients. One of them had contact to pet dogs and two came from the same region and the same time frame in 2016. Those three showed an identical PAI, but with decreased similarity and different length (8 kb) compared to the others. The lower similarity of the PAI of these three isolates, compared to that of the other isolates was probably also due to variation in the integrase following the tRNA-Arg CDS, resulting in higher similarity to integrase accession BAM26371 than to AKN76028, as annotated in the other samples’ PAIs (Additional file 2: Table S3). Furthermore, the PAI of those three samples showed one SNP in the pseudo-tRNA repeat. KL0556 was an outlier of the *C. ulcerans* clade and was located on an own branch of the tree (Fig. 3). The extracted sequence between tRNA-Arg and 200 bp downstream the *tox* gene was shorter (4.96 kb) compared to the other PAIs (7.7–8 kb) and the annotations detected in the other isolates were not present here. The region included four hypothetical proteins between the tRNA and the *tox* gene, with one of them being also a putative integrase, and additionally three SNPs in the pseudo-tRNA repeat.

Prophages in atypical NTTB *C. ulcerans* built a separate branch (Fig. 3, green clade) as it was also the case for DT (Fig. 1) and DtxR (Fig. 2). In all except one isolate their *tox*-embedding prophages were classified with questionable completeness status by the annotation software. This was mainly due to lacking overlap of the found CDS with the used databases of known prophage sequences, although a substantial extent of CDS was classified as phage sequences with specific functions. Most of the regions had a length of 31–33 kb or 43 kb (KL0396, KL0400). Only in KL0884 a prophage with a length of 51 kb was classified as intact by PHASTER. Despite its different length, the region overlapping with the shorter prophages of the other atypical NTTB *C. ulcerans* showed high similarity to them.

Generally, *tox*-overlapping prophages and PAIs showed different GC contents compared to the average GC content of the whole genome assemblies, as expected (Additional file 2: Table S3). In *C. diphtheriae* GC content was reduced by 1.2–1.5% and in atypical NTTB *C. ulcerans* by 1.5–3.8%. However, in phage regions of *C. ulcerans* a reduction by only 0.3–0.6% occurred, while in GC content reduction in PAI regions was substantially higher (5.6–5.8%). GC content of the shorter, different PAI in KL0556 was even reduced by 6.4%.

## Discussion

With the WGS data from different *Corynebacteriu*m species infecting different host organisms, we demonstrated pathogen species-specific clades of DT and *tox* gene-surrounding mobile, pathogenicity related genetic elements in toxigenic and NTTB isolates, as well as of DtxR in toxigenic, NTTB and non-toxigenic isolates (Table 1). For the *tox*-surrounding phage or PAI regions we found various branches, matching with those found in DT and DtxR. Surprisingly, a notable extent of heterogeneity of the *tox*-surrounding phage regions was found within *C. diphtheriae* for which research is done for decades. For example Chen and co-workers similarly found in 2008 that the lytic corynephage P1201 showed only 9% shared gene content with the only previously sequenced prophage BFK20 and that the mosaic-like genome of P1201 indicates extensive horizontal gene transfer among P1201, *Gordonia terrae* phage GTE5, mycobacteriophages and several regions of *Corynebacterium spp*. Genomes [13]. This finding may be true for further corynephages and at least partly explain the variations observed in our data. However, why other *Corynebacterium* species seem to be less prone to this variation than *C. diphtheriae* is not clear, but it may be associated with host species adaption and interaction.

Datasets of several *C. diphtheriae* isolates reanalysed in this study were part of a previous outbreak investigation of isolates from African refugees and could be classified in two outbreak clusters of epidemiological related samples by whole genome SNP profiles in the previous study. A third cluster detected back then included non-toxigenic samples that were not included in the current study [5]. Interestingly, in the current study the separation of the two included outbreak clusters could be reconstructed even on the level of DT and of the respective surrounding prophage, but not as clearly on DtxR level. Outbreak cluster 1 with isolates KL0633, KL0654, KL0663, KL0670 was affiliated to the above mentioned branch 3 of the *C. diphtheriae* clade and outbreak cluster 2 with isolates KL0623, KL0265, KL0652 to branch 2. Horizontal gene transfer in *C. diphtheriae* is an important factor for propagation of pathogenicity. However, this higher similarity of mobile pathogenicity-related elements in closely related strains indicates that important virulence features like DT and underlying prophages may at least be partly retained in bacterial populations and diverge along the evolutionary path with the bacterial core genome, potentially due to their advantages in host interaction or infectivity.

Additionally to the known *tox*-surrounding prophages, we found an alternative *tox*-surrounding PAI described in [7] in a majority of *C. ulcerans* isolates, which was not present in any isolate of the other analysed *Corynebacterium* species. Having a closer look at the CDS annotations of this PAI, which comprised several sequences with putative integrase activity, it could be assumed that this PAI also developed from a former not yet described prophage sequence. In general, the different pathogenicity-related features exist in zoonotic *C. ulcerans* across different host species*.* Interestingly, isolate KL0556 built an exception to all other *C. ulcerans* isolates, regarding its *tox*-surrounding PAI. The extracted region between tRNA-Arg and 200 bp downstream of *tox* was much shorter than the PAI of the other samples and showed different CDS annotations. The differences to the PAIs of the other *C. ulcerans* isolates with the same start and end annotations might be due to assembly problems, although the differences in annotated CDS sequences render this explanation of being a technical artefact very unlikely. The reason for it might also be evolution from previously more similar sequences. Another probably even more likely reason would be, especially regarding the differences of the integrase CDS, the pseudo tRNA-repeat and the lower GC content, that this isolate harbours another not yet described PAI, which might indicate the existence of a higher number of yet unknown corynephages.

Generally, besides *tox*-surrounding prophages or PAIs, we found a varying number of incomplete prophages in all our analysed strains. Similar observations have been made by Subedi et al. [18] in three different *C. ulcerans* strains, belonging to two different lineages, independent of the infected host organism. They linked the incompleteness of the prophages to the draft version of their NGS assembly data, but found that those prophages lead to significant diversity within *C. ulcerans*, consistent with earlier publications. Those assumptions concluded from the data of the three strains in [18] could very well be reconstructed in our larger dataset with more than 40 *C. ulcerans* isolates, and based on our data be expanded to at least *C. diphtheriae* and eventually also be postulated for *C. pseudotuberculosis.*

Additionally to the existence of various prophages within the strains the question of their propagation arises. Trost et al. described that *C. diphtheriae* comprises a stable, but open core genome of approx. 1600 genes (approximately 70% of the genome) being complemented by more variable gene content of 30%. Especially the variable content is to some extent subject to horizontal gene transfer. Furthermore, a recent study from Mansfield et al. [14] even discovered a DT-like protein family in bacterial lineages outside *Corynebacterium* genus, including *Austwickia* and *Streptomyces*. These genes show a certain degree of similarity to *tox*, but are surrounded by different loci. In all except one genus they did not discover any *dtxR*-like genes. The authors concluded that *tox*-like genes have not simply been exchanged between genera via corynephages in recent times, but either may have been acquired by independent mechanisms or the genomic evidence of the acquisition was lost over time.

Based on this knowledge and our results, which clearly demarcate clades between the different *Corynebacterium* species and show a certain degree of diversity within those clades, we conclude besides the fact that horizontal gene transfer via prophages is indeed likely to occur within species of the genus *Corynebacterium,* sequences may be based on different genetic backgrounds. Furthermore, once acquired elements also seem to evolve along with other virulence and pathogenicity related features within the different species. The alternative PAI exclusively present in *C. ulcerans* furthermore implies that DT transmission pathways between isolates may be different between human-pathogenic *C. diphtheriae* and the zoonotic species and contribute to the emerging pathogenic potential of the latter ones.

However, it has to be noted that despite the considerable evidence, our conclusions are based on results from short read assemblies with varying missing parts. Finishing of representative genomes which can be achieved by third generation sequencing technologies in future studies can strengthen the significance of our conclusions.

## Conclusion

With our phylogenetic analyses of DT, DtxR and *tox* gene-embedding mobile elements from WGS data of 91 toxigenic and 46 non-toxigenic isolates of different pathogenic *Corynebacterium* species, we were able to detect four nearly species-specific clades and verify previous postulations of species-specific DT differences based on a meaningful sample set. The clear clade-like separation of *tox* gene and surrounding genetic elements and the slight intra-clade variations even allow grouping of some related sample clusters. Furthermore a PAI, previously detected in a single isolate only could be classified as a general feature in a substantial number of zoonotic *C. ulcerans* across different hosts. These observations imply that the pathogenicity related features have been acquired a considerable time ago, by different and maybe independent events. They may furthermore correlate with host and environmental niche adaption in the different *Corynebacterium* species. Furthermore the features have probably been retained through evolution of strains due to their striking advantages in host interaction and infectivity. This conclusion on the important and potent pathogenicity factor DT and surrounding mobile elements gives a good example of how pathogenicity in *Corynebacterium spp.* could generally be established and propagated. This can also be of considerable value for research on related pathogenic bacteria. Furthermore, the findings are of special value for public health as the identified differences will be reflected in pathogen-host-interaction and may at least partly explain differences in disease manifestation, progression or treatment success. For example reduced immunity against strains with different corynephages and toxin types after vaccination with standard toxoid may occur. Furthermore, *C. ulcerans* is increasingly identified in human infections. These and especially the still rare but consistently occurring cases of pharyngeal diphtheria, caused by toxigenic *C. ulcerans* which are more often found in wound infections, may be associated with the differences in pathogenicity factors and an expanded host reservoir. Therefore, these topics may also be of reasonable interest for public health related applied research in the future.

## Methods

### Aim and design of the study

We aimed to investigate if differences in DT, its regulator DtxR and the *tox* gene-surrounding pathogenicity-related elements between different *Corynebacterium* species can be observed on a regular basis when a larger sample set is analysed and which phylogenetic conclusions regarding the DT-mediated pathogenicity of medically relevant corynebacteria can be drawn from the results. Therefore, we performed phylogenetic analyses with translated versions of the WGS-generated respective genes and genetic regions.

### Isolates selection

For phylogenetic comparison of DT, *tox* gene-surrounding pathogenicity related elements and DtxR, isolates from different *Corynebacterium* species or their previously generated whole genome sequencing (WGS) data were selected from the culture collection of the German Consiliary Laboratory on Diphtheria. Toxigenic isolates from different years, geographic regions and hosts were selected and complemented with non-toxigenic isolates from the same bacterial and host species for comparison (*C. diphtheriae*: 17 toxigenic, 34 non-toxigenic; typical *C. ulcerans*: 18 toxigenic, 21 non-toxigenic *tox* gene-bearing (NTTB), 7 non-toxigenic; atypical *C. ulcerans* from a wildlife cluster: 33 NTTB; *C. pseudotuberculosis*: 2 toxigenic, 5 non-toxigenic). For DT alignment 39 published DT sequences from *C. diphtheriae* (*N* = 22) and *C. ulcerans* (*N* = 17), originating from humans (*C. diphtheria* and *C. ulcerans*) or from dogs, moles and owls (*C. ulcerans*), were downloaded from NCBI and included (Additional file 1: Table S1).

### Bacteriological analyses

Bacteriological species identification was performed using MALDI-TOF MS analysis (microflex LT Mass Spectrometer, MALDI Biotyper™; Bruker Daltonics, MBT 7311 MSP library, Bremen, Germany) as described in [19]. The presence of *tox* gene was investigated by real-time PCR [20] and positive PCR results were phenotypically confirmed by a modified Elek test [21].

### Next generation sequencing and data analyses

Whole genome sequencing (WGS) of the isolates was performed on an Illumina MiSeq with 2 × 250 bp paired-end reads (Illumina, San Diego, CA, USA), after DNA isolation on the Promega Maxwell system and library preparation by Nextera XT, as reported previously [17].

Bioinformatics analyses started with quality control per sequencing run and sample using Illumina SAV software [22] and fastQC [23], respectively. Species identity and absence of contaminations from other species was checked with kraken [24]. Reads were trimmed for quality and from short, truncated reads with trimmomatic [25]. De novo assemblies were done with SPAdes assembler [26]. Assembly quality was checked with QUAST [27]. Contigs were ordered with the mauve ContigOrderer tool [28], along genbank-formatted reference genomes of toxigenic *C. diphtheria* NCTC 13129 (accession BX248353), *C. ulcerans* FRC58 (accession CP0011913.1) and *C. pseudotuberculosis* C231 (accession NC_017301). Reordered contigs were concatenated to continuous input files for subsequent annotation steps. Annotation of the draft genomes was done with prokka [29] using the default database and an additional *Corynebacterium* genus database, built with the prokka_database_maker script [30], based on NCBI-deposited sequence data from 305 tax IDs as database input (Additional file 3). Prophage detection, annotation and phage sequence extraction was performed with PHASTER [31] using the PHASTER url API.

Translated protein sequences of DT and DtxR were extracted from the annotated genbank-formatted draft genomes with open source python scripts [32] with the respective locus tag as identifier. For subsequent steps 39 NCBI-derived DT sequences were included (accession numbers in Additional file 1: Tables S1).

Extracted DT, DtxR and phage sequences were aligned using muscle [33] and maximum likelihood (ML) trees were built with FastTree, using the default Shimodaira-Hasegawa test on the three alternate topologies and 1000 resamples [34]. Similarity percentages of DT and DtxR alignments were calculated with CLC genomics workbench (Qiagen).

NGS raw datasets analysed during the current study are available in the NCBI sequence read archive (SRA) at https://www.ncbi.nlm.nih.gov/sra under BioProjects PRJNA416260 and PRJNA490531 (accession numbers in Additional file 1: Table S2).

## Additional files

Additional file 1: NCBI accession numbers of downloaded DT sequences (Table S1) and SRA accession numbers of analysed WGS data (Table S2). (DOCX 28 kb)

Additional file 2: Detected *tox*, *dtxR*, prophage and PAI coordinates, annotations and GC content (Table S3): For each of the 137 isolates, coordinates of *tox* and *dtxR* genes are given along with their species affiliation and their *tox* status analysed by qPCR and modified Elek test. All detected prophage sequences and PAIs are shown one line per region. For prophages and PAIs, information about region type and their overlapping or non-overlapping behavior in regard to the *tox* gene is indicated. For detected prophages, results from the prophage annotation software PHASTER [31] are given. For the alternative PAI, genomic start coordinates of the individual CDS sequences and structural annotations are indicated as well. Average GC content of the draft genome and the specific prophage/PAI regions is given. (XLSX 88 kb)

Additional file 3: Tax IDs used for the generation of the *Corynebacterium spp.* annotation database in prokka [29]. (DOCX 21 kb)

## Abbreviations

CDS: Coding sequence
DT: Diphtheria toxin
DtxR: Diphtheria toxin regulator
*dtxR*: Diphtheria toxin regulator gene
DUF: Domain of unknown function
HTH: Helix-turn-helix (motif)
ML: Maximum likelihood
MLST: Multi locus sequence typing
NGS: Next generation sequencing
PAI: Pathogenicity island
SNP(s): Single Nucleotide Polymorphism(s)
*tox*: Diphtheria toxin gene
WGS: Whole genome sequencing
