# Supplementary material for: NGS-based phylogeny of diphtheria-related pathogenicity factors in different Corynebacterium spp. implies species-specific virulence transmission
Source: BMC Microbiol. 2019 Feb 1;19:28. doi: 10.1186/s12866-019-1402-1 (PMC6359835; doi:10.1186/s12866-019-1402-1)
Supplement: Supplementary file 1 — NCBI accession numbers of downloaded DT sequences (Table S1) and SRA accession numbers of analysed WGS data (Table S2). (DOCX 28 kb) [file 12866_2019_1402_MOESM1_ESM.docx]

**NGS-based phylogeny of diphtheria-related pathogenicity factors in different *Corynebacterium spp.* implies species-specific virulence transmission - Additional file 1**

**Supplementary Table S1: Accession Numbers of NCBI derived DT sequenced included in alignment:**

| **Protein Accession (GenBank)** | **Gene accession (GenBank)** |
| --- | --- |
| AOU74567.1 | KX703000.1 |
| AOU74566.1 | KX702999.1 |
| AOU74565.1 | KX702998.1 |
| AOU74564.1 | KX702997.1 |
| AOU74563.1 | KX702996.1 |
| AOU74562.1 | KX702995.1 |
| AOU74561.1 | KX702994.1 |
| AOU74560.1 | KX702992.1 |
| AOU74559.1 | KX702991.1 |
| AOU74558.1 | KX702990.1 |
| AND74678.1 | KU248821.1 |
| AND74677.1 | KU248820.1 |
| AND74676.1 | KU248819.1 |
| AND74675.1 | KU248818.1 |
| AND74674.1 | KU248817.1 |
| AMP42520.1 | KU869775.1 |
| AMP42519.1 | KU869774.1 |
| BAU21072.1 | AB926015.1 |
| BAU21071.1 | AB926014.1 |
| BAU21070.1 | AB926013.1 |
| BAU21069.1 | AB926012.1 |
| AAN28948.1 | AY141013.1 |
| BAG06867.1 | AB304278.1 |
| BAO18778.1 | AB828261.1 |
| BAL14546.1 | AB602359.1 |
| BAL14545.1 | AB602358.1 |
| BAL14544.1 | AB602357.1 |
| BAL14543.1 | AB602356.1 |
| BAL14542.1 | AB602355.1 |
| AAW22870.1 | AY703827.1 |
| AAN28949.1 | AY141014.1 |
| BAH57338.1 | AB498872.1 |
| BAG06869.1 | AB304280.1 |
| BAL14541.1 | AB602354.1 |
| BAL14540.1 | AB602353.1 |
| BAK14371.1 | AB610405.1 |
| ACR57083.1 | FJ858272.1 |
| BAG06868.1 | AB304279.1 |
| AAV70486.1 | AY820132.1 |

**Supplementary Table S2: SRA accession numbers of whole genome sequencing data of *Corynebacterium spp.* isolates analysed in this study:**

| **Sample ID** | **Bioproject** | **SRR accession** | **Biosample** |
| --- | --- | --- | --- |
| 08-1143-CB1 | PRJNA490531 | SRR7825397 | SAMN10039575 |
| KL0126-cb2 | PRJNA490531 | SRR7825396 | SAMN10039576 |
| KL0160 | PRJNA490531 | SRR7825395 | SAMN10039577 |
| KL0182 | PRJNA490531 | SRR7825394 | SAMN10039578 |
| KL0183 | PRJNA490531 | SRR7825401 | SAMN10039579 |
| KL0194 | PRJNA490531 | SRR7825400 | SAMN10039580 |
| KL0195 | PRJNA490531 | SRR7825399 | SAMN10039581 |
| KL0199 | PRJNA490531 | SRR7825398 | SAMN10039582 |
| KL0246-cb3 | PRJNA490531 | SRR7825403 | SAMN10039583 |
| KL0251-cb4 | PRJNA490531 | SRR7825402 | SAMN10039584 |
| KL0252-cb5 | PRJNA490531 | SRR7825415 | SAMN10039585 |
| KL0259 | PRJNA490531 | SRR7825414 | SAMN10039586 |
| KL0260 | PRJNA490531 | SRR7825417 | SAMN10039587 |
| KL0262 | PRJNA490531 | SRR7825416 | SAMN10039588 |
| KL0263 | PRJNA490531 | SRR7825419 | SAMN10039589 |
| KL0264 | PRJNA490531 | SRR7825418 | SAMN10039590 |
| KL0265 | PRJNA490531 | SRR7825421 | SAMN10039591 |
| KL0266 | PRJNA490531 | SRR7825420 | SAMN10039592 |
| KL0269 | PRJNA490531 | SRR7825423 | SAMN10039593 |
| KL0276 | PRJNA490531 | SRR7825422 | SAMN10039594 |
| KL0315-cb6 | PRJNA490531 | SRR7825408 | SAMN10039595 |
| KL0318-cb7 | PRJNA490531 | SRR7825409 | SAMN10039596 |
| KL0330 | PRJNA416260 | SRR7039159 | SAMN07946374 |
| KL0345 | PRJNA490531 | SRR7825410 | SAMN10039597 |
| KL0349 | PRJNA490531 | SRR7825411 | SAMN10039598 |
| KL0350 | PRJNA490531 | SRR7825404 | SAMN10039599 |
| KL0355 | PRJNA416260 | SRR7039160 | SAMN07946375 |
| KL0360 | PRJNA416260 | SRR7039157 | SAMN07946376 |
| KL0371 | PRJNA416260 | SRR7039158 | SAMN07946377 |
| KL0372 | PRJNA416260 | SRR7039163 | SAMN07946378 |
| KL0374 | PRJNA490531 | SRR7825405 | SAMN10039600 |
| KL0377 | PRJNA416260 | SRR7039164 | SAMN07946379 |
| KL0382 | PRJNA490531 | SRR7825406 | SAMN10039601 |
| KL0386 | PRJNA490531 | SRR7825407 | SAMN10039602 |
| KL0387-cb8 | PRJNA490531 | SRR7825412 | SAMN10039603 |
| KL0392-cb9 | PRJNA490531 | SRR7825413 | SAMN10039604 |
| KL0394 | PRJNA490531 | SRR7825389 | SAMN10039605 |
| KL0395 | PRJNA490531 | SRR7825388 | SAMN10039606 |
| KL0396 | PRJNA490531 | SRR7825387 | SAMN10039607 |
| KL0400 | PRJNA490531 | SRR7825386 | SAMN10039608 |
| KL0401 | PRJNA490531 | SRR7825393 | SAMN10039609 |
| KL0433 | PRJNA490531 | SRR7825392 | SAMN10039610 |
| KL0434 | PRJNA416260 | SRR7039202 | SAMN07946380 |
| KL0438 | PRJNA416260 | SRR7039162 | SAMN07946381 |
| KL0442 | PRJNA490531 | SRR7825391 | SAMN10039611 |
| KL0451 | PRJNA490531 | SRR7825390 | SAMN10039612 |
| KL0459 | PRJNA490531 | SRR7825385 | SAMN10039613 |
| KL0461 | PRJNA416260 | SRR7039165 | SAMN07946382 |
| KL0468 | PRJNA490531 | SRR7825384 | SAMN10039614 |
| KL0472 | PRJNA490531 | SRR7825382 | SAMN10039615 |
| KL0475 | PRJNA490531 | SRR7825383 | SAMN10039616 |
| KL0476 | PRJNA416260 | SRR7039155 | SAMN07946383 |
| KL0479 | PRJNA416260 | SRR7039177 | SAMN07946384 |
| KL0483 | PRJNA490531 | SRR7825380 | SAMN10039617 |
| KL0497 | PRJNA490531 | SRR7825381 | SAMN10039618 |
| KL0501 | PRJNA490531 | SRR7825378 | SAMN10039619 |
| KL0507 | PRJNA416260 | SRR7039178 | SAMN07946385 |
| KL0515 | PRJNA490531 | SRR7825379 | SAMN10039620 |
| KL0522 | PRJNA416260 | SRR7039175 | SAMN07946386 |
| KL0540 | PRJNA490531 | SRR7825376 | SAMN10039621 |
| KL0541 | PRJNA490531 | SRR7825377 | SAMN10039622 |
| KL0547 | PRJNA490531 | SRR7825374 | SAMN10039623 |
| KL0556 | PRJNA490531 | SRR7825375 | SAMN10039624 |
| KL0557 | PRJNA416260 | SRR7039176 | SAMN07946387 |
| KL0565 | PRJNA490531 | SRR7825371 | SAMN10039625 |
| KL0581 | PRJNA490531 | SRR7825370 | SAMN10039626 |
| KL0585 | PRJNA416260 | SRR7039181 | SAMN07946388 |
| KL0598 | PRJNA490531 | SRR7825373 | SAMN10039627 |
| KL0599 | PRJNA416260 | SRR7039182 | SAMN07946389 |
| KL0603 | PRJNA490531 | SRR7825372 | SAMN10039628 |
| KL0613 | PRJNA490531 | SRR7825367 | SAMN10039629 |
| KL0615 | PRJNA490531 | SRR7825366 | SAMN10039630 |
| KL0623 | PRJNA490531 | SRR7825369 | SAMN10039631 |
| KL0625 | PRJNA490531 | SRR7825368 | SAMN10039632 |
| KL0631 | PRJNA490531 | SRR7825365 | SAMN10039633 |
| KL0633 | PRJNA490531 | SRR7825364 | SAMN10039634 |
| KL0638 | PRJNA490531 | SRR7825354 | SAMN10039635 |
| KL0652 | PRJNA490531 | SRR7825355 | SAMN10039636 |
| KL0654 | PRJNA490531 | SRR7825356 | SAMN10039637 |
| KL0655 | PRJNA490531 | SRR7825357 | SAMN10039638 |
| KL0663 | PRJNA490531 | SRR7825358 | SAMN10039639 |
| KL0670 | PRJNA490531 | SRR7825359 | SAMN10039640 |
| KL0675 | PRJNA416260 | SRR7039215 | SAMN07946395 |
| KL0676 | PRJNA416260 | SRR7039214 | SAMN07946396 |
| KL0678 | PRJNA416260 | SRR7039213 | SAMN07946397 |
| KL0691 | PRJNA416260 | SRR7039220 | SAMN07946398 |
| KL0693 | PRJNA416260 | SRR7039219 | SAMN07946399 |
| KL0698 | PRJNA416260 | SRR7039218 | SAMN07946400 |
| KL0707 | PRJNA490531 | SRR7825360 | SAMN10039641 |
| KL0709 | PRJNA490531 | SRR7825361 | SAMN10039642 |
| KL0713 | PRJNA416260 | SRR7039224 | SAMN07946402 |
| KL0747 | PRJNA416260 | SRR7039223 | SAMN07946403 |
| KL0759 | PRJNA416260 | SRR7039161 | SAMN07946404 |
| KL0762 | PRJNA416260 | SRR7039167 | SAMN07946405 |
| KL0768 | PRJNA416260 | SRR7039168 | SAMN07946406 |
| KL0770 | PRJNA416260 | SRR7039169 | SAMN07946407 |
| KL0773 | PRJNA490531 | SRR7825362 | SAMN10039643 |
| KL0774 | PRJNA490531 | SRR7825363 | SAMN10039644 |
| KL0785 | PRJNA490531 | SRR7825346 | SAMN10039645 |
| KL0788 | PRJNA416260 | SRR7039166 | SAMN07946408 |
| KL0796 | PRJNA490531 | SRR7825345 | SAMN10039646 |
| KL0798 | PRJNA416260 | SRR7039205 | SAMN07946409 |
| KL0811 | PRJNA416260 | SRR7039153 | SAMN07946410 |
| KL0812 | PRJNA416260 | SRR7039156 | SAMN07946411 |
| KL0818 | PRJNA490531 | SRR7825344 | SAMN10039647 |
| KL0825 | PRJNA490531 | SRR7825343 | SAMN10039648 |
| KL0832 | PRJNA490531 | SRR7825342 | SAMN10039649 |
| KL0840 | PRJNA490531 | SRR7825341 | SAMN10039650 |
| KL0846 | PRJNA490531 | SRR7825340 | SAMN10039651 |
| KL0853 | PRJNA490531 | SRR7825339 | SAMN10039652 |
| KL0867 | PRJNA490531 | SRR7825353 | SAMN10039653 |
| KL0870 | PRJNA490531 | SRR7825352 | SAMN10039654 |
| KL0876 | PRJNA490531 | SRR7825438 | SAMN10039655 |
| KL0880 | PRJNA490531 | SRR7825439 | SAMN10039656 |
| KL0882 | PRJNA490531 | SRR7825436 | SAMN10039657 |
| KL0883 | PRJNA490531 | SRR7825437 | SAMN10039658 |
| KL0884 | PRJNA490531 | SRR7825442 | SAMN10039659 |
| KL0886 | PRJNA490531 | SRR7825443 | SAMN10039660 |
| KL0887 | PRJNA490531 | SRR7825440 | SAMN10039661 |
| KL0927 | PRJNA490531 | SRR7825441 | SAMN10039662 |
| KL0938 | PRJNA490531 | SRR7825434 | SAMN10039663 |
| KL0941 | PRJNA490531 | SRR7825435 | SAMN10039664 |
| KL0950 | PRJNA490531 | SRR7825427 | SAMN10039665 |
| KL0956 | PRJNA490531 | SRR7825426 | SAMN10039666 |
| KL0957 | PRJNA490531 | SRR7825429 | SAMN10039667 |
| KL0968 | PRJNA490531 | SRR7825428 | SAMN10039668 |
| KL1003 | PRJNA490531 | SRR7825431 | SAMN10039669 |
| KL1006 | PRJNA490531 | SRR7825430 | SAMN10039670 |
| KL1007 | PRJNA490531 | SRR7825433 | SAMN10039671 |
| KL1008 | PRJNA490531 | SRR7825432 | SAMN10039672 |
| KL1009 | PRJNA490531 | SRR7825425 | SAMN10039673 |
| KL1010 | PRJNA490531 | SRR7825424 | SAMN10039674 |
| KL1015 | PRJNA490531 | SRR7825347 | SAMN10039675 |
| KL1017 | PRJNA490531 | SRR7825348 | SAMN10039676 |
| KL1025 | PRJNA490531 | SRR7825349 | SAMN10039677 |
| KL1058 | PRJNA490531 | SRR7825350 | SAMN10039678 |
| KL1059 | PRJNA490531 | SRR7825351 | SAMN10039679 |
