# Supplementary material for: NGS-based phylogeny of diphtheria-related pathogenicity factors in different Corynebacterium spp. implies species-specific virulence transmission
Source: BMC Microbiol. 2019 Feb 1;19:28. doi: 10.1186/s12866-019-1402-1 (PMC6359835; doi:10.1186/s12866-019-1402-1)
Supplement: Supplementary file 3 — Tax IDs used for the generation of the Corynebacterium spp. annotation database in prokka [29]. (DOCX 21 kb) [file 12866_2019_1402_MOESM3_ESM.docx]

**NGS-based phylogeny of diphtheria-related pathogenicity factors in different *Corynebacterium spp.* implies species-specific virulence transmission - Additional file 3**

**List of used Tax IDs for generation of additional Corynebacterium annotation database:**

649754, 1697, 1705, 257309, 698962, 698966, 698972, 698973, 698969, 698968, 698963, 698970, 698965, 698967, 698971, 698964, 1162302, 1166015, 1206106, 1206107, 1717, 1450521, 1450520, 1450522, 1450523, 1720349, 2014537, 2014578, 1806053, 340322, 196627, 1075088, 1079988, 1204414, 1232381, 1232383, 1198672, 1310161, 1718, 1232384, 765874, 681645, 679896, 889513, 935298, 1087453, 935697, 1087454, 1087452, 1074485, 1117942, 1089446, 1087451, 1168865, 1161911, 1719, 1121353, 1724, 1725, 1223513, 858619, 1727, 28028, 35755, 1121366, 35756, 1121357, 35757, 927655, 1381111, 1121368, 862512, 525260, 144183, 38286, 585529, 306537, 525262, 38289, 38301, 1223512, 38302, 525264, 553206, 1224164, 1410654, 38305, 548477, 548478, 39791, 1348662, 42817, 553204, 43765, 566549, 553207, 1121367, 43769, 525268, 43770, 504474, 1267754, 43771, 53374, 1035195, 945711, 945712, 996634, 1258583, 1408268, 65058, 1006579, 1200352, 95617, 99807, 1451189, 108486, 1121354, 1287475, 136857, 146827, 196164, 156976, 156978, 1110505, 1285583, 645127, 161879, 525263, 1121365, 161895, 161896, 161899, 1161099, 1310164, 168810, 548476, 169292, 185761, 187491, 1437874, 191610, 1431546, 1121362, 225326, 1121356, 1121355, 662755, 1401064, 1224162, 401472, 1203190, 441500, 1121364, 1121370, 1111729, 1121363, 1121360, 571915, 1224163, 1125779, 702953, 702957, 702959, 702967, 702969, 863239, 1050174, 1072256, 1078764, 558173, 1203557, 1203558, 1203559, 1203560, 1203561, 1203562, 1203563, 1203564, 1203566, 1203567, 1203619, 1203622, 1203624, 1203625, 1203627, 1203632, 1223515, 1437875, 1408189, 1232427, 1384076, 1404245, 931089, 1414719, 1487956, 1544413, 1544416, 1581050, 1581056, 1581065, 1581066, 1581069, 1581072, 1581088, 1581089, 1581090, 1581098, 1581103, 1581113, 1581115, 1581116, 1581117, 1581119, 1581121, 1581123, 1581126, 1581134, 1581137, 1581138, 1581139, 1603887, 1610489, 1652495, 1686286, 1715006, 1715013, 1715017, 1715027, 1715030, 1715039, 1715042, 1715045, 1715083, 1715093, 1715096, 1715114, 1715130, 1715135, 1715136, 1715157, 1715160, 1715169, 1715171, 1715177, 1715187, 1715193, 1715203, 1715213, 1715215, 1720192, 1720193, 1737425, 1739252, 1739255, 1739256, 1739264, 1739285, 1739289, 1739291, 1739292, 1739295, 1739312, 1739324, 1739351, 1739353, 1739361, 1739364, 1739379, 1739382, 1739392, 1739400, 1739403, 1739416, 1739424, 1739436, 1739439, 1739447, 1739464, 1739478, 1739480, 1739482, 1739489, 1739494, 1739497, 1739500, 1739502, 1739514, 1739534, 1739536, 1739540, 1739545, 1739548, 1816684, 1834153, 1852380, 1852390, 1904962, 1906332, 1906333, 1906334, 1979527, 2026255, 2029400, 2029401
